# Late initiation of antenatal care and associated factors among pregnant women in Addis Zemen primary hospital, South Gondar, Ethiopia

**DOI:** 10.1186/s12978-019-0745-2

**Published:** 2019-05-31

**Authors:** Haileab Fekadu Wolde, Adino Tesfahun Tsegaye, Malede Mequanent Sisay

**Affiliations:** Department of Epidemiology and Biostatistics, Institute of Public Health, College of Medicine and Health Sciences, University of Gondar, Gondar, Ethiopia

**Keywords:** Antenatal care, Late initiation, Pregnant women, Risk factors

## Abstract

**Background:**

Antenatal care (ANC) is special care for pregnant women with the aim of preventing, detecting and treating health problems in both the fetus and mother. Early ANC attendance promotes early detection and treatment of complications which result in proper management during delivery and puerperium. However, the majority of pregnant women in Ethiopia initiate their ANC late. Therefore, this study aimed to assess the prevalence of late initiation of ANC and its associated factors among attendants in Addis Zemen primary hospital.

**Method:**

An institution-based cross-sectional study was conducted at Addis Zemen primary hospital from February 7 to June 122,018. The systematic random sampling technique was employed to select 369 pregnant women who attended ANC in the hospital. Data cleaning and analysis was done using SPSS version 25 statistical software. Descriptive statics and bi variable and multivariable logistic regression models were employed to assess the magnitude and factors associated with late initiation of ANC defined as making the first visit after 12 weeks of gestation.

**Result:**

This study indicated that 52.5% of the attendants initiated ANC late. The multivariable logistic regression analysis showed that being housewife (Adjusted odds ratio (AOR) = 2.85, 95% CI: 1.36, 5.96), self-employment (AOR = 2.38, 95% CI: 1.12, 5.04), travel expenses (AOR = 1.72, 95% CI: 1.05, 2.81), poor knowledge about ANC (AOR = 2.98, 95% CI: 1.78, 5.01) and unplanned pregnancy (AOR = 2.31, 95% CI: 1.28, 4.16) were significantly associated with late ANC initiation.

**Conclusion:**

The prevalence of late ANC initiation remains a major public health issue in Ethiopia. The major factors for being late were found to be poor knowledge, being housewife, and self-employment, travel expenses and unintended pregnancy. District and zonal health offices should work to create awareness about the importance of early initiation of ANC, make the service closer to the community and increase contraceptive utilization.

## Plain English summery

Antenatal care is a special care that is provided for pregnant women with the aim of improving the health of the unborn baby and the mother. According to WHO recommendation, every pregnant women should book ANC before 12 weeks of gestation. Early initiation of ANC has the benefit of early detection and treatment of complications during pregnancy. However, the majority of women in Ethiopia initiate ANC late. Therefore, the objective of this study was to assess the magnitude of late initiation of ANC and factors affecting it among ANC attendants in Addis Zemen primary hospital.

Of the total 364 participants, 191 started their follow up late. Being housewife, self employment, travel cost, poor knowledge about ANC and unintended pregnancy were the factors that increased the likelihood of initiating ANC late.

In conclusion, late initiation of ANC was high in the study area. Poor knowledge level, being housewife and self-employment, travel expenses and unintended pregnancy were significantly associated with late initiation of ANC. creating about the importance of early initiation of ANC, making the service closer to the community and increasing contraceptive utilization would help to decrease the number of mothers who start ANC late.

## Background

Maternal mortality reduction remains a priority agenda in the new sustainable development goals (SDGs 3). However, it remains the global challenge with 275,288 deaths due to pregnancy and related complications in 2015 [1]. The burden is high in developing countries, accounting for 99% of the global maternal deaths in 2015, with the Sub-saharan Africa region including Ethiopia contributing 66% of the mortality [2, 3]. Most of the causes of maternal deaths are preventable, detectable, and treatable. Therefore, immediate action is needed to meet the ambitions of SDG 2030 for eliminating preventable causes of maternal death with a special attention to Sub- saharan Africa [4, 5] Antenatal care is one of the key strategies for reducing maternal and neonatal morbidity and mortality directly through the detection and treatment of pregnancy related illness, or indirectly through detection of women at risk of complications of delivery and ensuring that they deliver in a suitably equipped facility. During ANC, health providers monitor and identify risk factors related to poor maternal and birth outcomes. Once problems are identified, providers can initiate appropriate medical and educational interventions to reduce the risks for maternal-neonatal morbidity and mortality [6, 7]. However, early ANC visit is very low (24%) in low income countries compared with 81.9% in developed countries [8].

ANC services, especially the first visit, includes essential screening for health conditions such as human immunodeficiency virus (HIV) and Syphilis; for HIV-infected pregnant women, the maximum benefit of antiretroviral therapy (ART) to prevent mother-to-child transmission (PMTCT) of HIV requires early presentation to the health system. Screening for syphilis should be offered to all pregnant women at an early stage of ANC because treatment of syphilis is beneficial to the mother and the fetus. In pregnant women with early untreated syphilis, 70 to 100% of the infants will be infected and one-third will be stillborn. Furthermore, iron supplementation and immunizations, such as Tetanus Toxoid (TT), given during pregnancy can be life-saving for both mothers and infants if it is initiated at an early stage of pregnancy [9–12]. Early initiation of ANC also has a big role in reducing bad perinatal outcomes like preterm birth, low birth weight [13], and jaundice [14]. The aims of early ANC booking are identification of complications or risk factors for complications which enable early interventions to alleviate or mitigate the effects of such complications on mothers and unborn babies [15].

Since 2003, the Ethiopian government has been deploying specially trained new cadres of community-based workers called Health Extension Workers (HEWs). HEWs are expected to spend 75% of their time in outreach activities by going from house to house in their respective kebeles. They are trained on how to provide care to pregnant women throughout pregnancy, birth and post natal period [16].

Factors identified for late initiation of ANC include lack of education, poor knowledge about ANC, unplanned pregnancy, high cost ofANC, low income, multi parity, unemploymentand having history of abortion, [10, 17–23].

Even though WHO (World Health Organization) recommends that the first ANC visit be within the first 12 weeks of pregnancy [7], studies conducted in different parts of Ethiopia showed very low coverage, and that most of the women who started their follow up late [24–27]. In order to decrease child and maternal mortality, it is crucial to know the time of the first ANC visit of pregnant women and factors affecting it. Therefore, this study aimed to measure the magnitude and factors associated with late initiation of ANC at Addis Zemen primary hospital.

## Methods

### Study design and setting

**Fig. 1:**
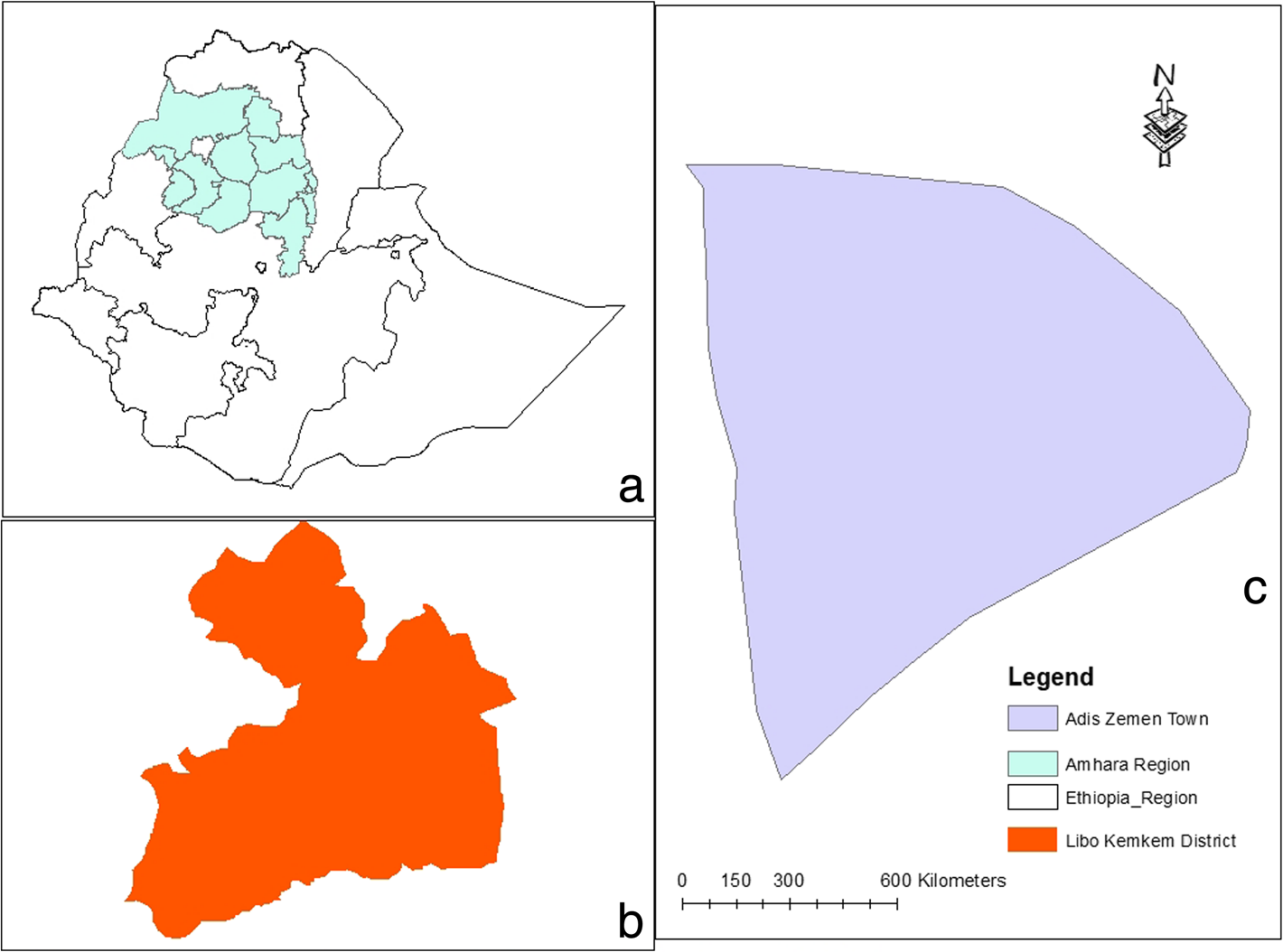
Location of the study area. **a** Ethiopia. **b** Libo-Kemkem. **c** Addis Zemen

An institution based cross-sectional study was conducted from February 7 to June 12, 2018.The study was conducted atAddis Zemen primary hospital, located in Libo kemkem district South Gondar, Amhara regional state, 658 KM northwest of Addis Ababa. The district had an estimated population of 198, 951 of whom 100,951 were male and 97,423 female [28]. Addis Zemen town had 28,003 male and 28,913 are female inhabitants. The town and its suburbs, have a primary hospital, a health center and 8 health posts. The hospital has opened2016, provides general care in medical, surgical, gynecology or obstetrics and pediatrics wards (Fig. 1).

### Study population and sampling procedure

The study populations of this study were all pregnant mothers who visited Addis Zemen primary hospital during the study period. Mothers mentally and physically incapable of being interviewed were excluded. The sample size was determined by using the formula for the estimation of a single population proportion with an assumption of 95% confidence interval, 5% margin of error, and 64.6% of expected proportion of late booking for ANC [11]. To compensate for the non-response rate, 10% of the determined sample size was added. A sample of 364 pregnant women who were attending ANC Clinic were selected using systematic random sampling technique with a skip interval of 2 was used, and the first subject was selected by using the lottery method.

### Variables data collection procedures

The outcome variable of the study was late booking for ANC which was defined as booking ANC after 12 weeks of gestation [12]. The explanatory variables included socio-demographic factors (age, religion, ethnicity, marital status, educational level, monthly income, occupation and travel cost); obstetric factors (gravidity, parity, history of abortion, type of pregnancy, history of ANC, timing of the first ANC visit from previous pregnancy, history of cesarean section (CS), history of still birth, problem in the last pregnancy and history of child death); enabling factors (distance from health institution, cost of travel), and need factor which includes knowledge towards ANC. Knowledge was measured using nine questions with “yes”/“no” responses. Then the composite score was dichotomized using the median (five) as a cutoff value so a score equal to or above the median value showed “good knowledge” and a score below the median value showed “poor knowledge”.

Data was collected using a structured interviewer-administered questionnaire that was prepared based on the study objectives. The data collection tool was translated into the local language (Amharic). Four health officers with BSc degrees and two Msc graduates in public health were involved as data collectors and supervisors, respectively. To control data quality, a one day training was given to data collectors on the aim of the study and on how to select participants to collect the data as per the data collection tool by the principal investigator. The tool was pre-tested on 5% of the actual sample out of the study area, and the filled copies were checked by the supervisors daily.

### Data processing and analysis

The data were entered into EPI info version 7.0 and transferred to SPSS version 25 for analysis. Chi-square test was done for all categorical independent variables to check the assumptions. Variance inflation factor (VIF) was used to check multi-collinearity. Descriptive statistics, like frequencies and percentages were used to describe the categorical independent variables. The binary logistic regression model was fitted as a primary method of analysis. Variables found to have < 0.2 *p*-values in the bi-variate logistic regression analysis were entered in to the multivariable logistic regression model. Goodness of fit of the model was assessed by using the Hosmer- Lemeshow goodness of fit test. Variables with less than 0.05 p - values in the multivariable model were considered significantly associated with the dependent variable. Odds ratio (OR) with a 95% confidence interval were computed to show the strength of associations.

## Results

### Socio demographic characteristics of the study participants

A total of 364 participants were involved in the study with a response rate of 94%. The majority of the participants, 282(77.5%), 20–35 age groups with the minimum and maximum ages of 18 and 44 years, respectively. Nearly 85% (309) of the participants were married, while 352(96.7%) and 257(70.6%) were Amhara and Orthodox Christian, respectively. Of the participants, 160(44%) were housewives and 87(23.9%) self-employed Besides, 113(31%) were with no formal education, and only 69(19%) were diploma or above graduates. More than half of the participants, 209(57.4%), had ETB > 1000 monthly income. Over one-third, 131(36%), of the participants had no travel expens to and from ANC facility (Table 1).

**Table 1:** Socio demographic characteristics of pregnant women who were attending ANC at Addis Zemen primary hospital, February –June 2018 (*n* = 364)

Variable	Time of ANC initiation		Total n (%)
	Early n (%)	Late n (%)	
Age			
< 20	12 (6.9)	17 (8.9)	29 (8)
20–35	128 (74)	154 (80.6)	282 (77.5)
> 35	33 (19.1)	20 (10.5)	53 (14.6)
Ethnicity			
Amhara	167 (96.5)	185 (96.9)	352 (96.7)
Other^a^	6 (3.5)	6 (3.1)	12 (3.3)
Religion			
Orthodox	118 (68.2)	139 (72.8)	257 (70.6)
Muslim	47 (27.2)	39 (20.4)	86 (23.6)
Protestant	7 (4)	13 (6.8)	20 (5.5)
Catholic	1 (0.6)	0 (0)	1 (0.3)
Marital status			
Single	15 (8.7)	20 (10.5)	35 (9.6)
Married	152 (87.9)	157 (82.2)	309 (84.9)
Divorced/widowed	6 (3.5)	14 (7.3)	20 (5.5)
Educational level			
No formal education	47 (27.2)	66 (34.6)	113 (31)
Primary (1–8)	44 (25.4)	67 (35.1)	111 (30.5)
Secondary and preparatory	32 (18.5)	39 (20.4)	71 (19.5)
Diploma and above	50 (28.9)	19 (9.9)	69 (19)
Occupation			
Government employed	47 (27.2)	18 (9.4)	65 (17.9)
Self employed	37 (21.4)	50 (26.2)	87 (23.9)
House wife	69 (39.9)	91 (47.6)	160 (44)
Other^b^	20 (11.6)	32 (16.8)	52 (14.3)
Income			
< 400 ETB	19 (11)	15 (7.9)	34 (9.3)
400–1000 ETB	44 (25.4)	77 (40.3)	121 (33.2)
> 1000 ETB	110 (63.6)	99 (51.8)	209 (57.4)
Transport cost			
< 3 ETB	41 (23.7)	28 (14.7)	69 (19)
3–9 ETB	35 (20.2)	33 (17.3)	68 (18.7)
> 9 ETB	43 (24.9)	53 (27.7)	96 (26.4)
Did not pay	54 (31.2)	77 (40.3)	131 (36)

Other^a^- Oromo/Tigre/ Gurage, other^b^-Daily laborer/ NGO/ farmer/ student. ETB: Ethiopian Birr

### Obstetric history of the study participants

Of the total respondents, 128(35.2%) were primigravida and 167(70.8%) multiparous. Of these, 200(84.7%) reported that they had ANC experience during their previous pregnancy. Among respondents who had had an experience of ANC follow up 129(64.5%) visited ANC clinic for the first time after 12 weeks of gestation in the previous pregnancy. Out of 31(13.1%) respondents who had history of abortion, 25 (80.64%) were spontaneous.. Ninety-four (25.8%) of the pregnancies were unintended. Of these, 64 came late for ANC for the current pregnancy. The majority of the respondents, 171 (72.5%) faced no problems during their last pregnancies, and only 8(5%) had history of still births. Twenty-six (11%) of respondents had history of CS delivery (Table 2).

**Table 2:** Obstetric history of pregnant women who were attending ANCat Addis Zemen primary hospital, February –June 2018 (*n* = 364)

Variable	Time of ANC initiation		Total n (%)
	Early n (%)	Late n (%)	
Gravidity (*n* = 364)			
Primigravida	55 (31.8)	73 (38.2)	128 (35.2)
Multigravida	118 (68.2)	118 (61.8)	236 (64.8)
Parity (*n* = 236)			
Primiparous	37 (31.4)	32 (27.1)	69 (29.2)
Multiparus	81 (68.6)	86 (72.9)	167 (70.8)
Abortion history (*n* = 236)			
Yes	17 (14.4)	14 (11.9)	31 (13.1)
No	101 (85.6)	104 (88.1)	205 (86.9)
Planned pregnancy (*n* = 364)			
Yes	143 (82.7)	127 (66.5)	270 (74.2)
No	30 (17.3)	64 (33.5)	94 (25.8)
Alive child (*n* = 234)			
0	1 (0.9)	2 (1.7)	3 (1.3)
1	37 (31.6)	32 (27.4)	69 (29.5)
2–4	69 (59)	78 (66.7)	147 (62.8)
> 4	10 (8.5)	5 (4.3)	15 (6.4)
Child death (*n* = 222)			
Yes	2 (1.8)	4 (3.7)	6 (2.8)
No	112 (98.2)	104 (96.3)	216 (97.3)
Still birth(*n* = 160)			
Yes	2 (2.9)	6 (6.7)	8 (5)
No	68 (97.1)	84 (93.3)	152 (95)
Problem in the last pregnancy (*n* = 236)			
Yes	36 (30.5)	29 (24.6)	65 (27.5)
No	82 (69.5)	89 (75.4)	171 (72.5)
CS history (*n* = 236)			
Yes	13 (11)	13 (11)	26 (11)
No	105 (89)	105 (89)	210 (89)
History of ANC (*n* = 236)			
Yes	102 (86.4)	98 (83.1)	200 (84.7)
No	16 (13.6)	20 (16.9)	36 (15.3)
ANC initiation time for the previous pregnancy (*n* = 200)			
≤ 12 weeks	47 (46.1)	24 (24.5)	71 (35.5)
> 12 weeks	55 (53.9)	74 (75.5)	129 (64.5)

### Timing of the current first ANC visit and knowledge towards ANC

**Fig. 2:**
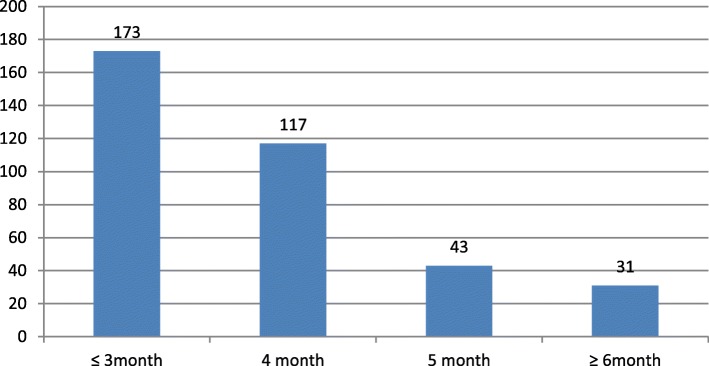
Timing of first ANC visit for pregnant mothers who were attending ANC in Addis Zemen primary hospital, February–June 2018

Of the total respondents, 173(47.5%) had their first ANC initiation in 12 weeks of conception and 191(52.5%) after 12 weeks (Fig. 2).

From the total respondents 247(67.9%) and 241 (66.2%) perceived that ANC was highly important for them and for the fetus respectively. From the total study participants, 143(39.3%) thought the appropriate time to book first ANC visit was before 12 weeks. From the total, 139(38.2%) thought ≥8 visits were required for ANC. One hundred ninety six (53.8%) respondents perceived that mothers needed nutritional supplementation during pregnancy. Regarding the overall Knowledge of the mothers towards ANC 220(60.4%) had a good knowledge **(**Table 3**).**

**Table 3:** Knowledge towards ANC among pregnant mothers who were attending ANC at Addis Zemen primary hospital, February–June 2018 (*n* = 364)

Questions	Yes	No
Do you think ANC is importance for maternal health?	247 (67.9)	117 (32.1%
Do you think ANC is important for the fetus?	241 (66.2%)	123 (33.8%)
Do you think the appropriate time to begin ANC after conception is < 12 weeks?	143 (39.3%)	221 (60.7%)
Do you think a woman needs to go for ANC > 8 times?	139 (38.2%)	225 (61.8%)
Do you think a pregnant mother need to initiate ANC early only if she had complication in her previous pregnancy?	237 (65.1%)	127 (34.9%)
Do you think pregnant mother needs supplementation during her pregnancy?	196 (53.8%)	168 (46.2%)
Do you know maternal conditions risk for the fetus	137 (37.6%)	227 (62.4%)
Dou you think appropriate ANC follow up can prevent disease transmission from mother to child?	308 (84.6%)	56 (15.4%)
Dou you think appropriate ANC follow up can prevent erythroblastosis fetalis?	29 (8%)	335 (92%)

### Factors associated with late initiation of ANC

In the bivariate analysis, factors like age, occupation, monthly income, knowledge level, health education, husband support and whether the pregnancy was planned or not were significantly associated with late initiation of ANC. However, in the multivariable analysis, only variables like occupational status, travel cost, knowledge about ANC and whether the pregnancy was planned or not were found to be the independent predictors of late booking for ANC. The odds of booking ANC late was 2.85 times higher among housewives compared to mothers who were government employed keeping other variables constant (AOR = 2.85, 95% CI:1.36, 5.96). The odds of booking ANC late was 2.38 times higher among self-employed mothers compared to government employed keeping other variables constant (AOR = 2.38, 95% CI: 1.12, 5.04). The odds of booking ANC late increased by 72% among mothers who paid for transport to go to ANC service compared to those who did not pay keeping other variables constant (AOR = 1.72, 95% CI: 1.05, 2.81). The odds of being late for booking ANC was nearly three times higher among mothers who had poor knowledge of ANC as compared to mothers with good knowledge keeping other variables constant (AOR = 2.98, 95% CI: 1.78, 5.01). The odds of being late for booking ANC was 2.31 times higher among mothers who had unintended pregnancy compared to mothers with planned pregnancy keeping other variables constant (AOR = 2.31, 95% CI: 1.28, 4.16) (Table 4).

**Table 4:** Multivariable logistic regression of predictors to late ANC initiations among pregnant women at Addis Zemen primary hospital, February –June 2018 (*n* = 364)

Variable	Crud OR (95% CI)	Adjusted HR (95% CI)	*P*-value
Age (years)			
> 35	1	1	
< 20	2.3 (0.9,5.9)	1.6 (0.50, 5.10)	0.426
20–35	1.2 (1.1, 3.6)	2.0 (0.95, 4.29)	0.066
Marital status			
Married	1	1	
Other^a^	1.6 (0.9, 2.8)	0.93 (0.46, 1.86)	0.822
Educational status			
Literate	1	1	
No formal education	1.4 (0.9, 2.2)	0.89 (0.48, 1.62)	0.696
Occupation			
Government employed	1	1	
Housewife	3.4 (1.8, 6.4)	2.85 (1.36, 5.96)	**0.005**
Self employed	3.5 (1.8, 7.0)	2.38 (1.12, 5.04)	**0.024**
Other^b^	4.2 (1.9, 9.1)	2.13 (0.87, 5.19)	0.096
Income/month			
> 1000 ETB	1	1	
< 400 ETB	0.9 (0.4, 1.8)	0.53 (0.23, 1.30)	0.163
400–1000 ETB	1.9 (1.4, 3.1)	1.51 (0.87, 2.60)	0.142
Transport cost			
Don’t pay	1	1	
Pay	1.5 (0.9, 2.3)	1.72 (1.05, 2.81)	**0.033**
Gravidity			
Multigravida	1	1	
Primigravida	1.3 (0.8, 2.0)	0.87 (0.50, 1.52)	0.626
Knowledge level			
Good	1	1	
Poor	3.6 (2.3, 5.6)	2.98 (1.78, 5.01)	**0.000**
Planned pregnancy			
Yes	1	1	
No	2.4 (1.4, 4)	2.31 (1.28, 4.16)	**0.005**
Health education			
Yes	1	1	
No	2.1 (1.3, 3.2)	1.67 (0.98, 2.85)	0.059
Advice about ANC			
Yes	1	1	
No	1.4 (0.9, 2.2)	0.87 (0.50, 1.50)	0.609
Husband support			
Yes	1	1	1
No	2.0 (1.2, 3.1)	1.31 (0.73, 2.34)	0.360

Other^a^-single/divorced/widowed, Other^b^-daily laborer/NGO/farmer/student, ANC: anti natal care, ETB: Ethiopian Birr

The boldface indicates significant associations with *p*-value < 0.05

## Discussion

This study mainly assessed the prevalence of late ANC initiation and associated factors among pregnant mother who attended ANC at Addis Zemen primary hospital. Different studies reported different risk factors for late initiation of ANC; our study assessed the socio demographic, obstetric, enabling, and need factors. As a result, factors like housewife status and self-employment, payment for travel, poor knowledge about ANC, and unintended pregnancy were found be significantly associated with late initiation of ANC.

World Health Organization recommends that pregnant mothers, especially those who are living in developing countries should start ANC in the first three months of pregnancy [12]. In our study however more than half of the respondents (52.5%) initiated ANC after the recommended time. This result is consistent with those other studies conducted in Adigrat (Ethiopia) [23], South Africa [10], Myanmar [29] and Gondar (Ethiopia) [30]. In this study he prevalence of booking ANC late was higher than those of studies done in Addis Ababa and Debre Markos, Ethiopia which showed 42 and 33.4% respectively [31, 32]. This could be due to the fact that, Addis Ababa is the capital of the country and the community might have better awareness and access to health service than people in other parts of the country. Another reason could be the classification of the outcomes because the study in Debre Markos classified mothers as late for booking ANC if they come after 16 weeks of gestation, while our study classified a mother as being late if she came after 12 weeks.. On the other hand, our finding was lower than the result of other studies done in Ambo,Ethiopia (86.8%) [24] Kembata Tembaro zone, Ethiopia (68.6%) [27], Gondar,Ethiopia (65%) [11], Southern Ethiopia (78.3%) [25], Tanzania (70.4%) [17], Zambia (72%) [22], Nigeria (82.6%) [33], East Wollega,Ethiopia (81.5%) [26], Gedio Zone,Ethiopia (64.6%) [19] and meta-analysis that used a polled data from studies done in Ethiopia (64%) [20]. This could be explained by the socio-cultural differences among the study populations. Another reason could be time differences between the studies because currently there is a better improvement in awareness about ANC and there is also good access to the health facilities than the past times in Ethiopia [34].

In this study, housewives and self-employed mothers had increased odds of booking ANC late than government employed mothers. This result is consistent with that of a study done in Tanzania [17] and showed that housewives were at increased odds of booking ANC late. The reason could be explained by the workload housewives shoulder in the house and the lack of time to go to health facilities. Another reason could be poor educational status because the majority did not have formal education. The possible reason for self-employed mothers to be late for ANC might be lack of time. In addition, mothers might be busy making money for the basic needs of their families. Therefore, even if they knew the appropriate time for the visit, they might be late because of their busy days. According to our study, the odds of booking ANC late increased by 72% among mothers who paid for transport to get to ANC service compared to those who did not pay. This result is consistent with a study done in Addis Ababa [18]. The possible reason could be that mothers, especially from rural areas are expected to travel long distance to reach health facilities; so they may need wait until they get money for transport.

In our study, 39.6% of the mothers had poor knowledge about ANC which was associated with 2.98 times increased odds of booking ANC late. This result is in line with those of studies conducted in Ethiopia [19, 20, 25] and Zambia [22]. This could be explained by the fact that mothers with poor knowledge may not have awareness about the correct time for booking and may not know the importance of ANC both for the mother and the fetus.

According to our study, mothers who had unintended pregnancy had 2.31 times increased odds of booking ANC late compared to mothers with planned pregnancy. This result is consistent with those of other studies done in different parts of Ethiopia [18, 21, 23, 27, 32, 35], Zambia [22] and South Africa [10]. The possible reason might be that mothers with unintended pregnancy may miss a support from their partners or families which may decrease their good health seeking behavior [36]. Another reason could be that women with unplanned pregnancies may initially attempt to deny their pregnancies to themselves and to conceal them from others. As a result, such women become less motivated to seek ANC early compared to women with planned pregnancies. Furthermore, unplanned pregnancies are also related to socio-cultural determinants of health-seeking behaviors, sexual violence, and barriers to access which may be associated with late initiations [10].

The limitation of this study was using only governmental health facilities because some pregnant women may visit private health facilities so these mothers were not included in our study. Besides, studies use different cutoff points to categorize women as late or otherwise, making it difficult to compare our findings with other studies.

## Conclusion

Late booking for ANC was high in Ethiopia. According to our study the factors for lateness were being housewife and self-employment, poor knowledge about ANC, paying for travel to go for ANC, and unintended pregnancy. The district and zonal health offices should work on creating awareness about the benefits of early initiation of ANC both for the mother and the fetus. On the other hand, awareness should be created about family planning utilization to prevent untended pregnancies. It is also better to make the service closer to mothers who need to travel long distances. Moreover, the government also needs to meet the WHO-recommendation for the accessibility of health facilities to pregnant women.

## Data Availability

The data upon which the result based could be accessed a reasonable request.

## Abbreviations

ANC: Antenatal Care
AOR: Adjusted odds ratio
ART: Anti retroviral treatment
BSc: Bachelor of sciences
CS: Ceserian section
EDHS: Ethiopian demographic health survey
ETB: Ethiopian Birr
HIV: Human immune deficiency virus
KM: Kilo meters
OR: Odds ratio
PMTCT: Prevent mother-to-child transmission
SPSS: Statistical package for social science
TT: Tetanus toxoid
VIF: Variance inflation factor
WHO: World Health Organization
